# Second primary cancers in patients with skin cancer: a population-based study in Northern Ireland

**DOI:** 10.1038/sj.bjc.6604842

**Published:** 2009-01-06

**Authors:** M M Cantwell, L J Murray, D Catney, D Donnelly, P Autier, M Boniol, C Fox, R J Middleton, O M Dolan, A T Gavin

**Affiliations:** 1 Cancer Epidemiology and Prevention Research Group, Centre for Clinical and Population Sciences, Queen's University Belfast, Belfast, Northern Ireland; 2 Queen's University Belfast, Northern Ireland Cancer Registry, Royal Victoria Hospital, Belfast, Northern Ireland; 3 Epidemiology Methods and Support group, International Agency for Research on Cancer, Lyon, France; 4 Department of Dermatology, Royal Victoria Hospital, Belfast, Northern Ireland

**Keywords:** skin cancer, melanoma, squamous cell, basal cell

## Abstract

Among all 14 500 incident cases of basal cell carcinoma (BCC), 6405 squamous cell carcinomas (SCC) and 1839 melanomas reported to the Northern Ireland Cancer Registry between 1993 and 2002, compared with the general population, risk of new primaries after BCC or SCC was increased by 9 and 57%, respectively. The subsequent risk of cancer, overall, was more than double after melanoma.

Melanoma and non-melanoma skin cancers (NMSC) (basal cell carcinoma (BCC) and squamous cell cancer (SCC)) show an increasing incidence worldwide (Jemal *et al*, 2001), including Northern Ireland (Hoey *et al*, 2007). There are limited epidemiological data concerning whether individuals with skin cancer are at an elevated risk of developing other malignancies. Tuohimaa *et al* (2007) combined data from 13 cancer registries and found 23 and 39% increased risks of several second primary cancers after a melanoma or NMSC, respectively. Decreases in risk of prostate (De Vries *et al*, 2007) and colorectal cancer (Soerjomataram *et al*, 2008) have been reported following a skin cancer, whereas a recent meta-analysis showed a significant reduction in colon cancer among patients with previous SCC (Grant, 2007).

We investigated the subsequent risk for any cancer in patients with BCC, SCC or melanoma. We also examined the reciprocal association for subsequent skin cancer after a primary colorectal cancer (as a validation exercise), as we hypothesised that patients with colorectal cancer, a cancer related to low vitamin D levels (Gorham *et al*, 2007), would have had a lower lifetime exposure to the sun and therefore have a lower risk of a subsequent skin cancer.

## Materials and methods

The Northern Ireland Cancer Registry (NICR) is a population-based registry that receives data routinely from 13 hospital administration systems, five pathology laboratories and eight radiology sites. It holds complete data on all neoplasms diagnosed within the province since the beginning of 1993. In addition, a partially complete database of tumours diagnosed between 1989 and 1992 is also held by the NICR. Patients with pathologically confirmed NMSC are registered according to the European Network of Cancer Registries’ guidelines for NMSC (Davies *et al*, 2007), which recommend that only the first occurrence of NMSC is registered based upon date of diagnosis and histological type (BCC or SCC). The latter is quality assured by NICR using all available information (such as primary site, patient history and general knowledge of tumour biology). Version 10 of the International Classification of Diseases was used to identify NICR registrations of NMSC (C44) and malignant melanoma (C43). Non-melanoma skin cancer cases registered with SNOMED codes between 80903 and 80953 were considered as BCC whereas those with SNOMED code 80703 or 80713 were considered SCC. Other types of NMSC were excluded from the analysis (1183 patients) due to the variety of histological types in the small number of patients.

In addition, the 1989–1992 database was used to eliminate from the 1993–2002 cohorts patients diagnosed with NMSC or melanoma during 1989–1992. A further 58 BCC, four SCC and two melanoma patients were also excluded as they were either diagnosed outside Northern Ireland (and could therefore not be followed for subsequent cancer risk) or were 100 years old or more when diagnosed. In total, 1837 melanoma, 6401 SCC and 14442 BCC patients were followed up to 31 December 2002 for a second primary neoplasm or death, the latter through linkage to death records held by the Registrar General's Office (GRO), Northern Ireland.

The outcome measures were classified according to ICD 10 as follows:

Any malignant neoplasm C00–C97; melanoma C43; SCC C44; BCC C44; lung C33 and C34; breast (female only) C50; cervix C53; uterus C54; ovary C56; prostate C61; oesophagus and stomach C15 and C16; colorectal C18–C21; haematological malignancies C81–C96; and non-Hodgkin's lymphoma C82–C85. Tobacco-related cancers were also grouped as one outcome variable and included ICD 10 codes C00–C16, C22, C25, C32–34, C53, C64–C68 and C92.

### Statistical analysis

Observed numbers of cancers and person-years at risk were calculated by sex and 5-year age group. Expected numbers of cancers were obtained by multiplying the age-specific numbers of person-years with the corresponding cancer incidence rates in Northern Ireland, adjusting for gender for the overall standardised incidence ratios (SIRs). Exact 95% confidence intervals (CIs) were calculated, assuming that the numbers of observed cases followed a Poisson distribution (Breslow and Day, 1987).

## Results

The absolute incidence of BCC was 86.6 cases per 100 000 person-years and the mean (s.d.) age at diagnosis was 66.7 (s.d. 12.9) years in men and 69.0 (s.d. 13.9) in women. Compared with the general population, overall, the BCC patients had a 9% increased incidence of new primary cancers, more than a two-fold increased risk of subsequent melanoma and 14% increased risk of SCC (Table 1).

The absolute incidence of SCC was 38.4 cases per 100 000 person-years and the mean (s.d.) age at diagnosis was 72.4 (11.4) years in men and 76.1 (12.0) years in women. In SCC patients, new primaries at any site were increased by more than 50% and the BCC incidence by more than two-fold (Table 2). Melanoma incidence was three times higher in men but not higher in women with an SCC and subsequent tobacco-related cancers were more likely in both sexes. Women with an SCC were less likely to have a subsequent breast cancer.

The absolute incidence of melanoma was 11.0 cases per 100 000 person-years. The mean (s.d.) age at diagnosis was 56.6 (18.3) years in men and 55.4 (20.3) years in women. Melanoma was followed by an increased risk of any subsequent cancer (SIR=2.06; 95% CI 1.73–2.39), melanoma (SIR=4.79; 95% CI 1.24–8.34), BCC (SIR=4.95; 95% CI 3.78–6.12) and SCC (SIR=2.50; 95% CI 1.24–3.77).

During the study period, the 9144 individuals registered with colorectal cancer showed, in contrast to our hypothesis, an increased risk of a second cancer (SIR=1.31; 95% CI 1.20–1.42) and an increased risk of BCC (SIR=1.36; 95% CI 1.11–1.61), a sun-related cancer. In addition, their subsequent risk of developing other cancers related to low vitamin D levels, such as prostate and breast cancer, was not increased (SIR=0.97; 95% CI 0.66–1.28 and SIR=0.89; 95% CI 0.55–1.23, respectively).

## Discussion

Our population-based study included all incident cases of BCC (14 422), SCC (6401) and melanoma (1837) reported to the Northern Ireland Cancer Registry between 1993 and 2002. Compared with the general population, the incidence of new primaries after BCC or SCC was increased by 9 and 57%, respectively. However, the subsequent risk of any cancer was more than double after melanoma.

Using NMSC, as a surrogate measure of long-term sun exposure, has suggested that exposure is associated with a lower risk of prostate cancer (de Vries *et al*, 2007). Our findings do not agree, and men with a BCC, SCC or melanoma did not have a decreased prostate cancer risk. An appreciable body of evidence suggests that the beneficial effect of continuous sun exposure on colorectal cancer risk is mediated through effects on the metabolism of vitamin D, as this can suppress cell proliferation, cancer development and metastasis (Gorham *et al*, 2007). We therefore hypothesised that colorectal cancer would be associated with a lower risk of other sun-related cancers (skin cancer) and a higher risk of developing cancers related to low vitamin D levels. In contrast, we found that they had an increased risk of a subsequent BCC and that their subsequent risk of prostate and breast cancer was not increased. Our results agree with those of Tuohimaa *et al* (2007), who reported that NMSC is only protective against subsequent solid cancers in sunny countries, which perhaps indicates a less variable vitamin D exposure and in turn less variable vitamin D status than in Northern, less sunny countries. In such countries, as Northern Ireland, vitamin D production from solar UVB is lower due to both shorter vitamin D production seasons and lower peak UVB doses (Grant, 2008).

We found that SCC patients were at a high risk of subsequent melanoma, confirming the results of Wassberg *et al* (1999), who reported an almost three-fold increase in melanoma risk. This is most likely due to a shared phenotype and environmental risk factors, although patients with NMSC are more likely to be screened for melanoma, and thereby have a spuriously high melanoma rate.

Subsequent tobacco-related cancers were more likely in SCC patients and this is not a surprising finding as cigarette smoking has been associated with a two-fold increased risk of SCC (Grodstein *et al*, 1995).

In general, an association between skin cancer and a second primary cancer could reflect shared aetiologic factors or biased ascertainment of new primaries as a result of increased surveillance (Schottenberg 1996). It should also be noted that individuals who are at a high risk of skin cancer may have other risk-taking behaviours related to their increase in risk of a second primary cancer such as smoking, dietary intake and physical activity levels.

There are some limitations to our study. Although based on the past 10 years data, the mean period of follow-up was only 4 years primarily due to the advanced age of patients at diagnosis, especially within the SCC cohort. Our study concerns a predominantly white population and so is not generalisable to other racial, ethnic groups. A general limitation to this and other registry-based investigations is the lack of information about individual UV exposures and other hypothesised risk factors

Several strengths of our study include the availability of data by NMSC subtype and its inclusion of data on all pathologically confirmed NMSC in Northern Ireland. Loss to follow-up is likely to be low; data from the GRO (Northern Ireland) estimate that less than 0.1% of the population migrated from the province per year during the 1990s (NISRA, 2008). Population-based studies of BCC are rare as hospitalisation is not required, prognosis is favourable and it is not routinely registered in most cancer registries.

Our results show that patients with a BCC, SCC or melanoma, compared with the general population, have an increased risk of developing a new primary cancer, especially melanoma in men. Our findings may partly reflect risk factors in common between these tumours, such as UV exposure and smoking, but they do not agree with some earlier reports of a decreased risk of prostate cancer after skin cancer perhaps mediated through vitamin D production.

## Figures and Tables

**Table 1:** New cancer in 14 442 patients with basal cell carcinoma of the skin in Northern Ireland: 1993–2002 (all ages)

		**Cancer in men**		**Cancer in women**		**Cancer in men and women**	
**Site**	**ICD 10**	***n***	**SIR (95% CI)**	***n***	**SIR (95% CI)**	***n***	**SIR (95% CI)**
All	C00–C97	674	**1.11** (1.03, 1.19)*	530	1.07 (0.98, 1.17)	1204	**1.09** (1.03, 1.16)*
All excluding non-melanoma skin cancer	C00–C97 ex C44	460	1.01 (0.92, 1.10)	391	1.08 (0.97, 1.19)	851	1.04 (0.97, 1.11)
Skin, melanoma	C43	19	**2.68** (1.47, 3.88)*	19	**2.11** (1.16, 3.07)*	38	**2.36** (1.61, 3.11)*
Skin, squamous cell	C44	214	**1.27** (1.10, 1.44)*	139	0.99 (0.83, 1.15)	353	**1.14** (1.02, 1.26)*
Lung	C33, C34	101	1.13 (0.91, 1.36)	51	1.19 (0.86, 1.51)	152	1.15 (0.97, 1.33)
Breast (female only)	C50			83	1.02 (0.80, 1.24)		
Cervix	C53			1			
Uterus	C54			19	1.52 (0.84, 2.21)		
Ovary	C56			10	0.61 (0.23, 1.00)		
Prostate	C61	107	1.17 (0.95, 1.39)				
Oesophagus and stomach	C15, C16	34	0.90 (0.60, 1.20)	24	1.04 (0.62, 1.46)	58	0.95 (0.71, 1.20)
Oesophagus	C15	12	0.91 (0.40, 1.43)	6	0.69 (0.14, 1.23)	18	0.82 (0.44, 1.20)
Stomach	C16	22	0.90 (0.52, 1.27)	18	1.26 (0.68, 1.84)	40	1.03 (0.71, 1.35)
Colorectal	C18–C21	67	0.90 (0.68, 1.11)	65	1.10 (0.83, 1.37)	132	0.99 (0.82, 1.16)
Colon	C18	44	0.92 (0.65, 1.19)	41	0.99 (0.69, 1.30)	85	0.95 (0.75, 1.15)
Rectum	C19–C21	23	0.85 (0.50, 1.20)	24	1.34 (0.80, 1.87)	47	1.05 (0.75, 1.35)
Haematological malignancies	C81–C96	45	1.24 (0.88, 1.60)	29	1.01 (0.64, 1.38)	74	1.14 (0.88, 1.40)
Non-Hodgkin's lymphoma	C82–C85	19	1.30 (0.72, 1.89)	17	1.20 (0.63, 1.77)	36	1.25 (0.84, 1.66)
							
Tobacco-related cancers	C00–C14, C15, C16, C22, C25, C32, C33, C34, C53, C64–C66, C67, C68, C92	193	0.95 (0.81, 1.08)	113	1.02 (0.83, 1.21)	306	0.97 (0.86, 1.08)

CI=confidence interval; SIR=standardised incidence ratio. Bold values indicate ^*^P<0.05.

**Table 2:** New cancer in 6401 patients with squamous cell carcinoma of the skin in Northern Ireland: 1993–2002 (all ages)

		**Cancer in men**		**Cancer in women**		**Cancer in men and women**	
**Site**	**ICD 10**	***n***	**SIR (95% CI)**	***n***	**SIR (95% CI)**	***n***	**SIR (95% CI)**
All	C00–C97	549	1.58 (1.44, 1.71)*	307	1.55 (1.38, 1.73)*	856	1.57 (1.46, 1.67)*
All excluding non-melanoma skin cancer	C00–C97 ex C44	352	1.34 (1.20, 1.48)*	174	1.21 (1.03, 1.39)*	526	1.29 (1.18, 1.40)*
Skin, melanoma	C43	12	3.13 (1.36, 4.91)*	3	0.86 (−0.11, 1.82)	15	2.04 (1.01, 3.08)*
Skin, basal cell	C44	197	1.97 (1.69, 2.25)*	133	2.19 (1.81, 2.56)*	330	2.05 (1.83, 2.27)*
Lung	C33, C34	47	0.94 (0.67, 1.21)	23	1.40 (0.83, 1.98)	70	1.05 (0.81, 1.30)
Breast (female only)	C50			17	0.58 (0.30, 0.86)		
Cervix	C53			4	2.89 (0.06, 5.72)		
Uterus	C54			4	0.92 (0.02, 1.81)		
Ovary	C56			3	0.52 (−0.07, 1.11)		
Prostate	C61	58	1.01 (0.75, 1.27)				
Oesophagus and stomach	C15, C16	32	1.48 (0.97, 2.00)	12	1.16 (0.50, 1.81)	44	1.38 (0.97, 1.78)
Oesophagus	C15	13	1.76 (0.80, 2.72)	6	1.52 (0.30, 2.73)	19	1.68 (0.92, 2.43)
Stomach	C16	19	1.34 (0.74, 1.94)	6	0.94 (0.19, 1.68)	25	1.21 (0.74, 1.69)
Colorectal	C18–C21	49	1.13 (0.81, 1.44)	23	0.93 (0.55, 1.32)	72	1.06 (0.81, 1.30)
Colon	C18	31	1.09 (0.71, 1.48)	15	0.87 (0.43, 1.32)	46	1.01 (0.72, 1.30)
Rectum	C19–C21	18	1.19 (0.64, 1.74)	8	1.07 (0.33, 1.82)	26	1.15 (0.71, 1.60)
Haematological malignancies	C81–C96	26	1.25 (0.77, 1.73)	16	1.37 (0.70, 2.04)	42	1.29 (0.90, 1.69)
Non-Hodgkin's lymphoma	C82–C85	9	1.14 (0.39, 1.88)	8	1.45 (0.44, 2.45)	17	1.26 (0.66, 1.87)
							
Tobacco-related cancers	C00–C14, C15, C16, C22, C25, C32, C33, C34, C53, C64–C66, C67, C68, C92	139	1.21 (1.01, 1.41)*	67	1.49 (1.13, 1.85)*	206	1.29 (1.11, 1.46)*

CI=confidence interval; SIR=standardised incidence ratio. Bold values indicate ^*^P<0.05.
